# Supplementary figures and images for: Mechano-biological and bio-mechanical pathways in cutaneous wound healing
Source: PLoS Comput Biol. 2023 Mar 9;19(3):e1010902. doi: 10.1371/journal.pcbi.1010902 (PMC10030043; doi:10.1371/journal.pcbi.1010902)

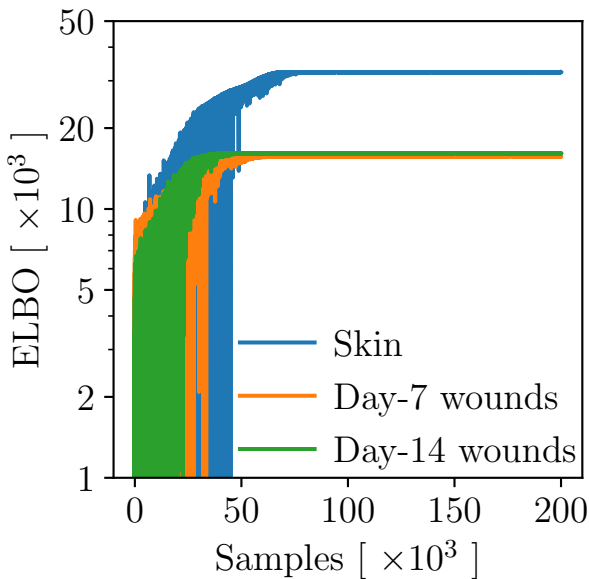

Supplement: S1 Fig — (PDF) [file pcbi.1010902.s001.pdf]

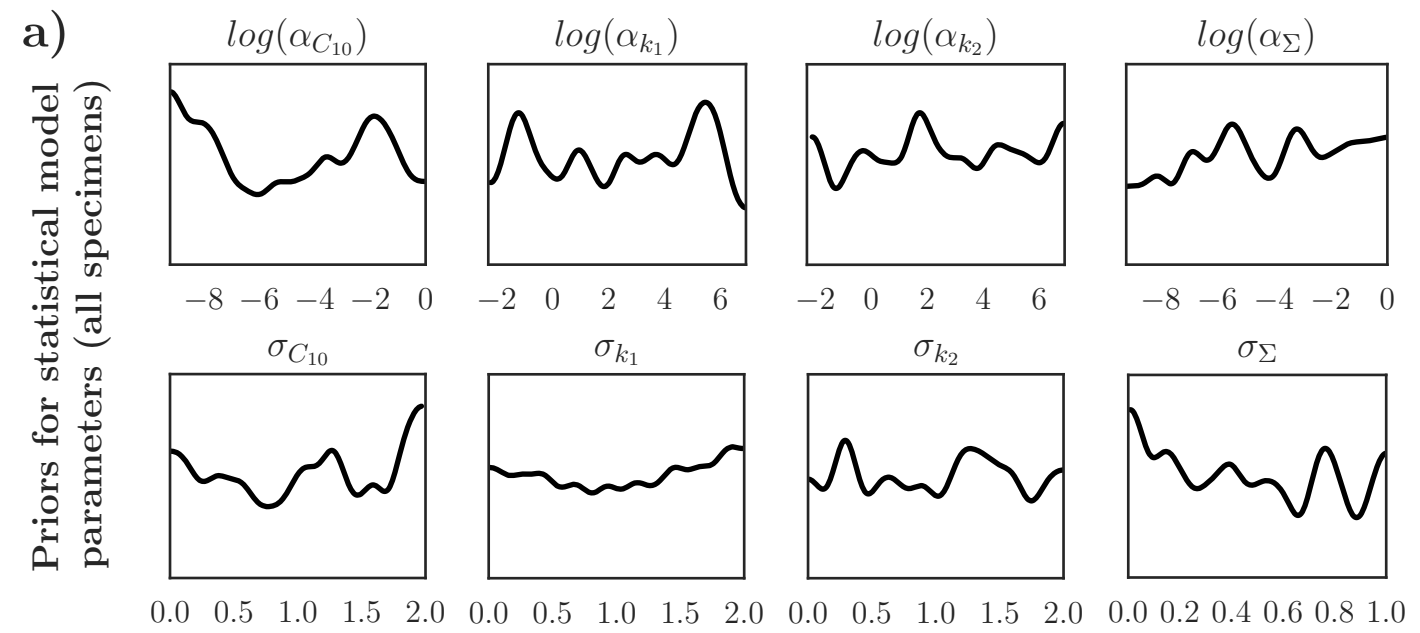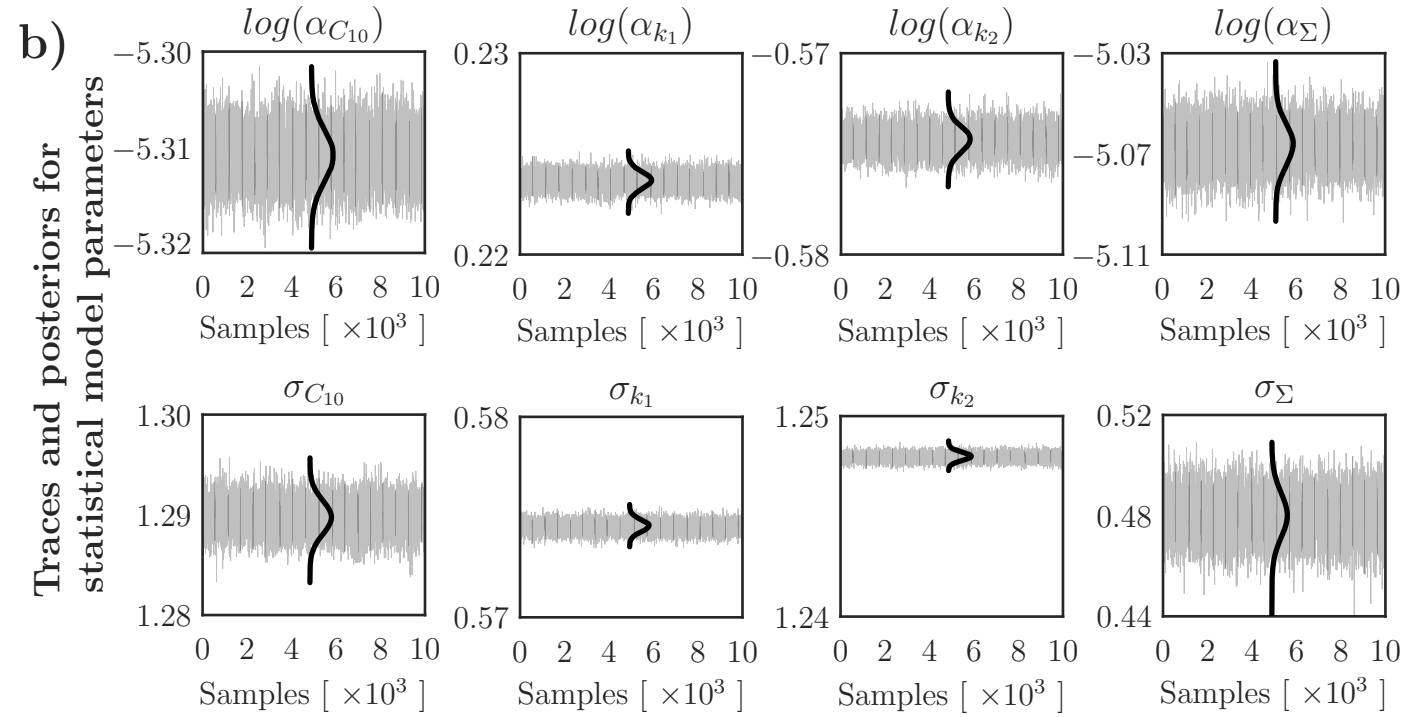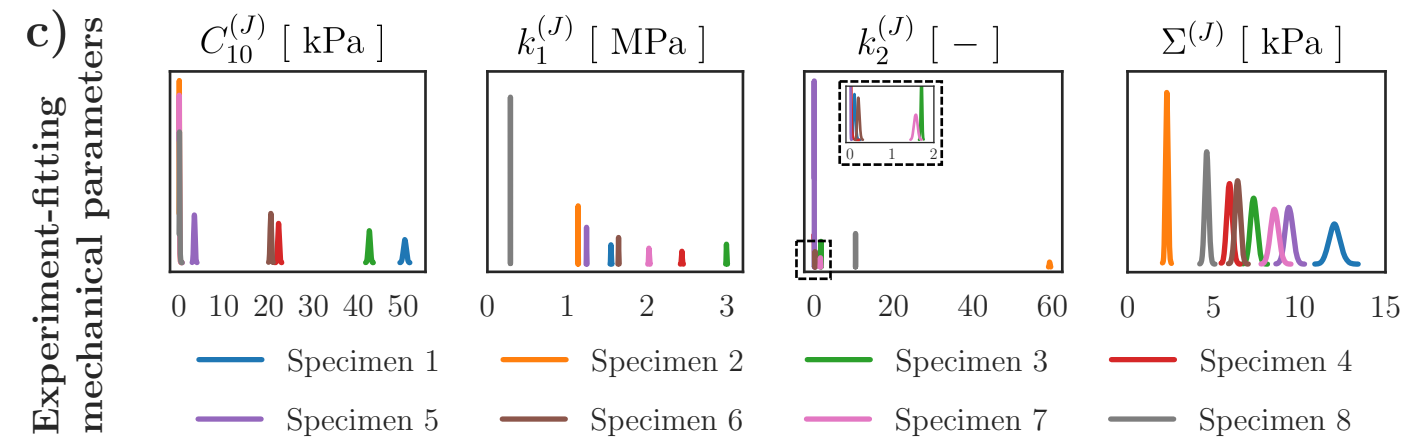

Supplement: S2 Fig — (PDF) [file pcbi.1010902.s002.pdf]

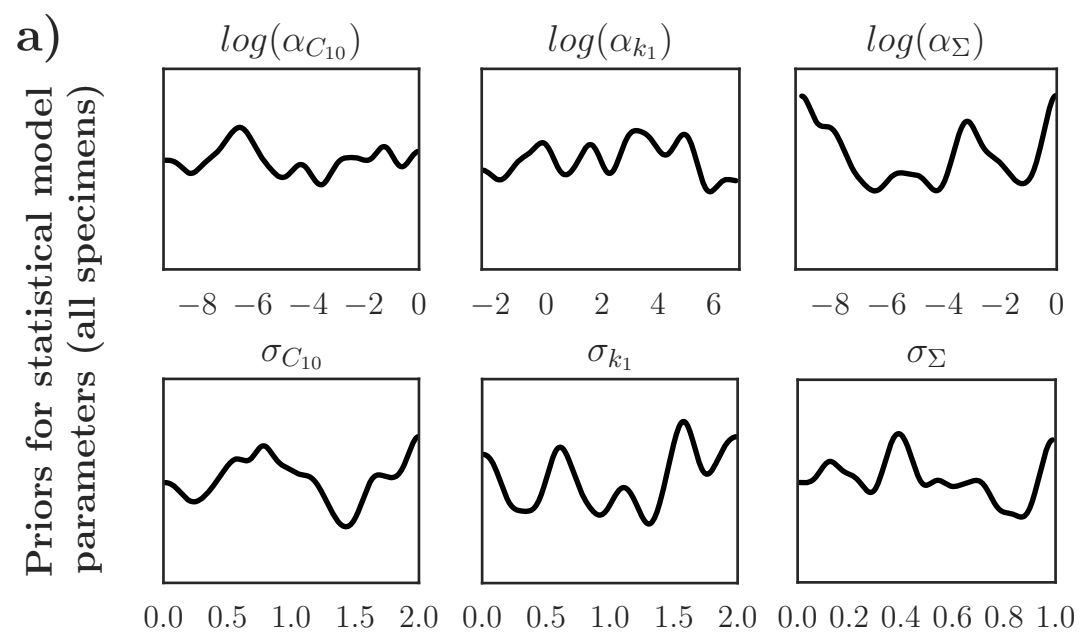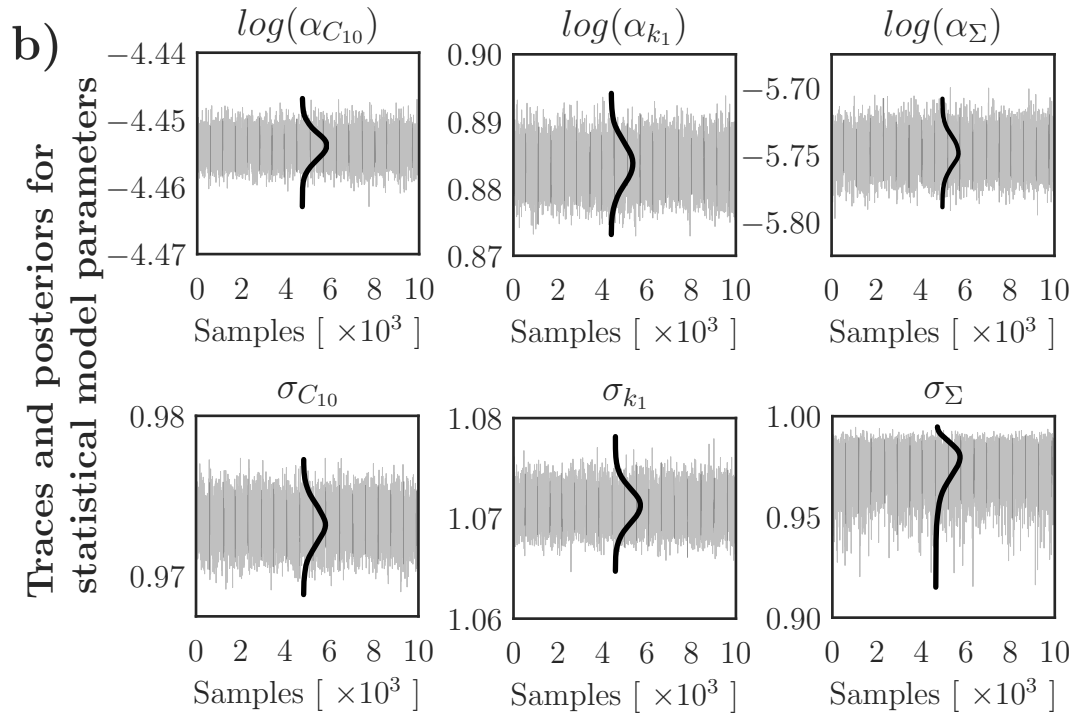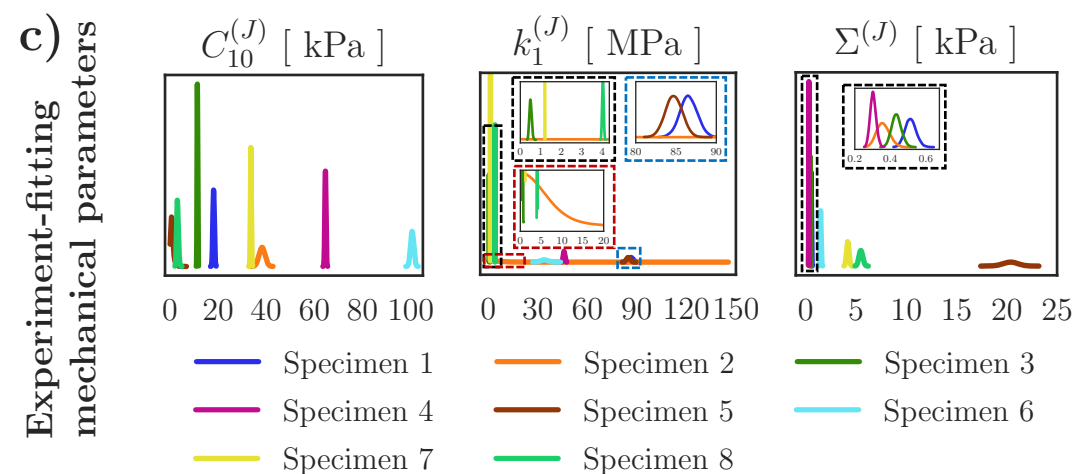

Supplement: S3 Fig — (PDF) [file pcbi.1010902.s003.pdf]

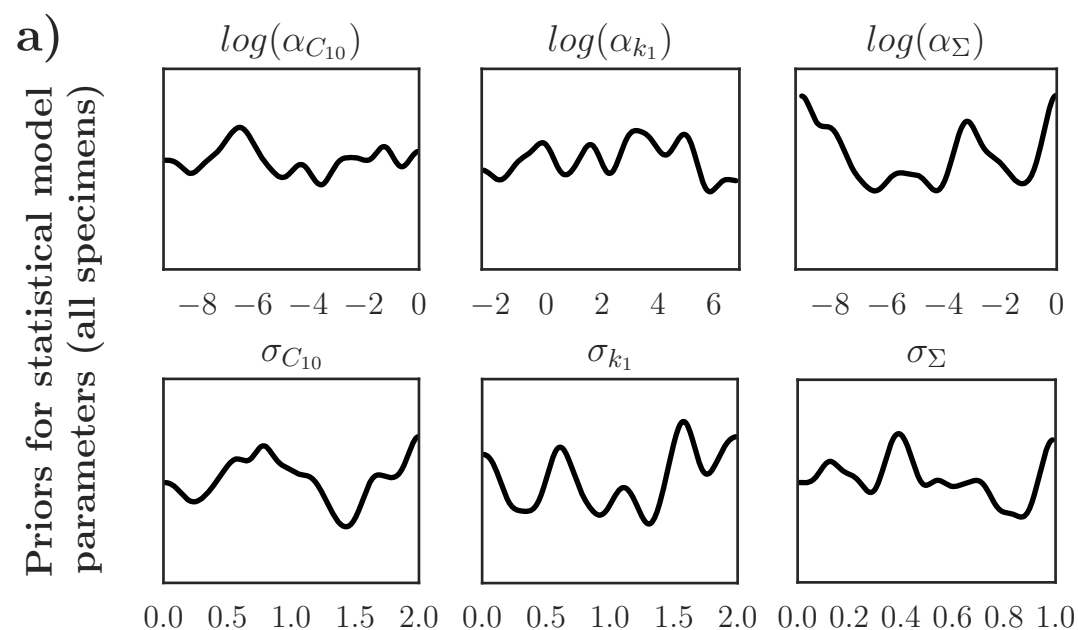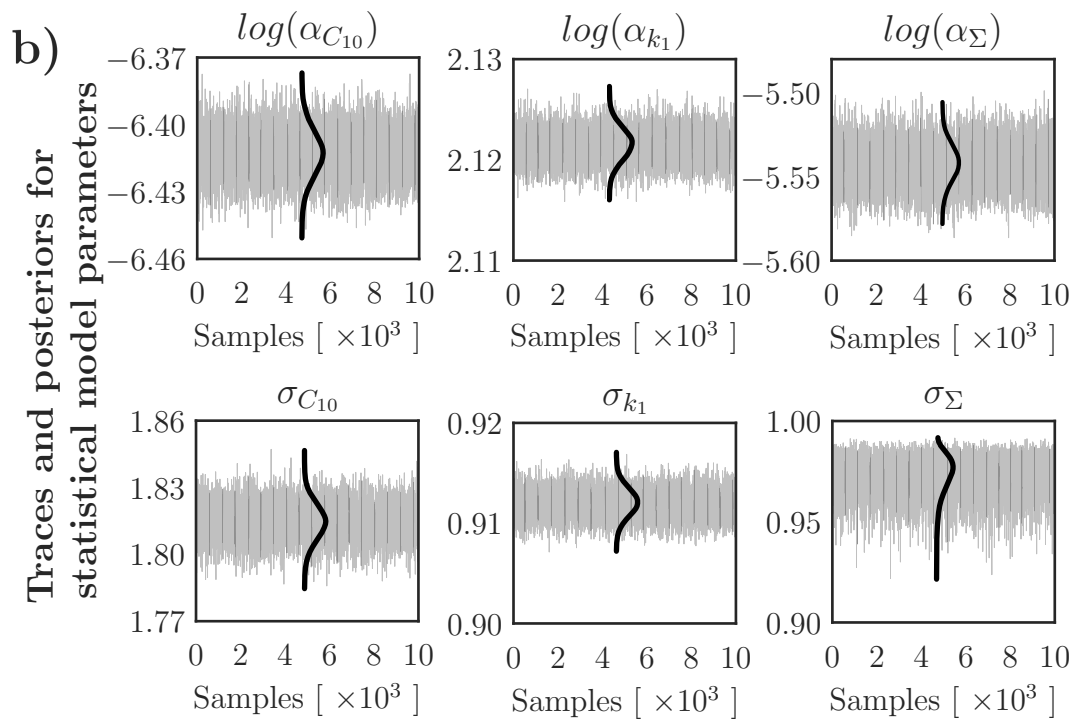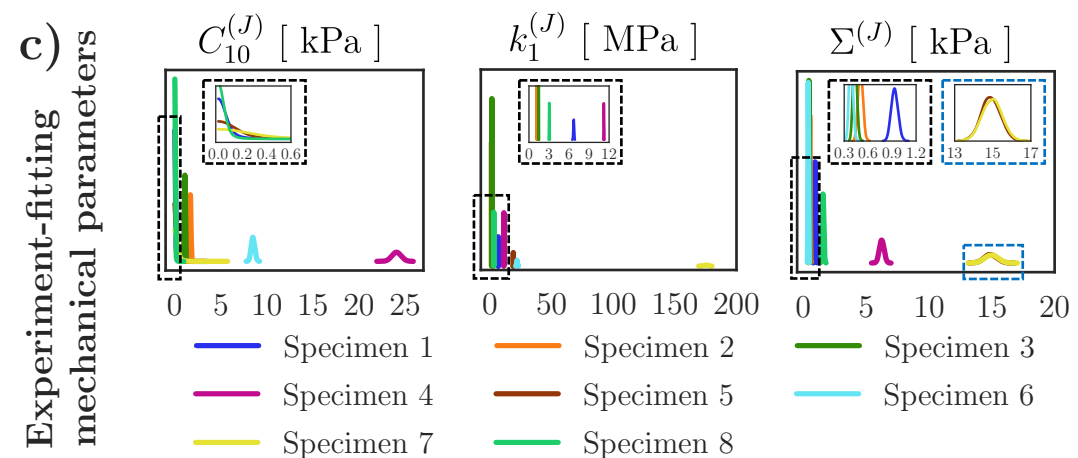

Supplement: S4 Fig — (PDF) [file pcbi.1010902.s004.pdf]
