# Supplementary material for: Mechano-biological and bio-mechanical pathways in cutaneous wound healing
Source: PLoS Comput Biol. 2023 Mar 9;19(3):e1010902. doi: 10.1371/journal.pcbi.1010902 (PMC10030043; doi:10.1371/journal.pcbi.1010902)
Supplement: S1 Appendix — (PDF) [file pcbi.1010902.s011.pdf]

S1 Appendix. Literature data for wound biochemistry and mechanobiology. To perform wide-range calibration of our custom FE model, we derive temporal evolutions of the biochemical fields c, ρ, and ϕ_c from published data on full-thickness excisional wounds in wild type mice (diameter: 3–8 mm). Unless numerical values are explicitly reported, we extract them from published charts using the web-based tool 'WebPlotDigitizer' [1]. For studies featuring measurement replicates, we focus on their average at each available time point. When multiple data from different studies are available for the same time point, we quantify and display their average and standard deviation (*cf.* Figs. 5-6 and Fig. A1.1).

To inform the temporal evolution of c, we consider TGF-β1 as a reliable indicator of the second inflammatory wave progression, owing to its undisputed role as the growth factor with the broadest spectrum of actions on cell activity within wound healing [2]. Focusing on experimental studies reporting the evolution of TGF-β1 throughout healing and the corresponding baseline values in unwounded tissue [2–5], we obtain the temporal evolutions in Fig. A1.1a.

For the cell content, ρ, we focus on previous studies reporting the number of fibroblasts in wounded and unwounded tissue [3,6], and obtain the temporal evolutions in Fig. A1.1b.

For the collagen content, ϕ_c, we consider measurements of wound hydroxyproline content [7–10] and obtain the corresponding collagen amount per mass of wet tissue according to the relation:

$$\phi_c^* = \frac{4H}{0.13\,\pi D^2 T P}, \tag{A1.1}$$

where H is the hydroxyproline mass measured in a tissue biopsy of diameter D, thickness T, and density P, while 0.13 is a conversion factor corresponding to the typical percentage of hydroxyproline within collagen [11]; in line with previous measurements, we take $P = 1.1$ mg mm^{-3} [12]. Using a similar approach, we also estimate the average collagen content in unwounded murine skin to be $\phi_c^{*,Skin} = 4.9\%$ [9, 13–18], which is remarkably close to recent experimental measurements (6.39% in wet mass) [19] and leads to the normalized wound collagen contents shown in Fig. A1.1c and in Figs.5,6.

For the dependence of cell activity on tissue deformation, we consider previous *in vitro* data on the proliferation of human patellar tendon fibroblasts under uniaxial

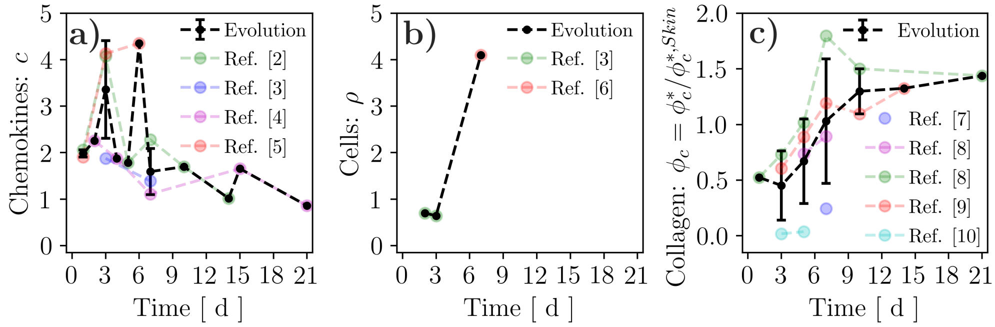

Figure A1.1. Temporal evolution of cytokines, c, cells, ρ, and collagen content, ϕ_c, according to several published studies (colored translucent dots connected by dashed lines showing trends). The data points for comparison with simulations are obtained by averaging information at corresponding time points, as obtained from different studies (black dots and error bars: mean ± standard deviation; dashed lines show trends).

cyclic stretch of increasing magnitude [20]. For comparability with our study, where we simulate tissue biaxial stretching from a physiological deformation state, we assume that the data in Ref. [20] can be interpreted as representative of fibroblast overproduction induced by a tissue overstretching $\theta^e/\theta^{ph} \sim (\lambda^{over}/\lambda^{ref})^2$, where λ^{over} are the uniaxial stretch values used in Ref. [20] and $\lambda^{ref} = 1$ is the corresponding reference value (no stretch). To determine the overexpression of ρ for an unwounded tissue under stretch, we consider the ODE system comprising Eqs. (5–7) and predict the values of c, ρ, and ϕ_c after 210 days of application of an areal deformation θ^e. Specifically, we set $\alpha = 0$ and select all parameters except for $\Omega^m = \Omega^m_\rho = \Omega^m_{\phi_c}$ according to S3 Table and S4Table. Since we are interested in stretch-mediated mechanosensing, we adopt the definition of $\hat{H}$ in Eq. (10). As shown in Fig. A1.2, increasing/decreasing the tissue stretch around its physiological value results in increased/decreased fibroblast production, in a way that depends on the value of Ω^m. Since values of Ω^m in the range of $0.005 - 0.02$ well capture the experimental data in Ref. [20], we select $\Omega^m = 0.01$ as the reference value for this study.

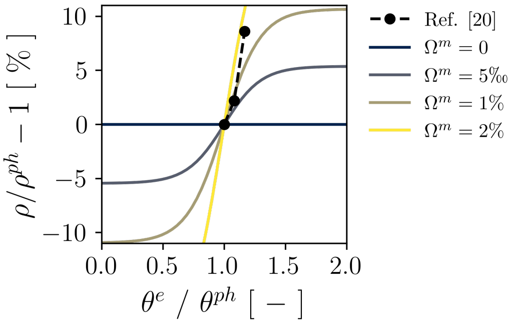

Figure A1.2. Dependence of fibroblast production on tissue stretch for alternative values of the coupling parameter Ω^m, in comparison with experimental data (black dots connected by a dashed line showing trend).

References

1. Rohatgi A. Webplotdigitizer: Version 4.5; 2021. Available from: `https://automeris.io/WebPlotDigitizer`.

2. Wang XJ, Han G, Owens P, Siddiqui Y, Li AG. Role of TGFβ-mediated inflammation in cutaneous wound healing. Journal of Investigative Dermatology Symposium Proceedings. 2006;11(1):112–117.

3. Reynolds L, Conti F, Lucas M, Grose R, Robinson S, Stone M, et al. Accelerated re-epithelialization in β3-integrin-deficient-mice is associated with enhanced TGF-β1 signaling. Nature medicine. 2005;11(2):167–174.

4. Komi-Kuramochi A, Kawano M, Oda Y, Asada M, Suzuki M, Oki J, et al. Expression of fibroblast growth factors and their receptors during full-thickness skin wound healing in young and aged mice. Journal of Endocrinology. 2005;186(2):273–289.

5. Ishida Y, Gao JL, Murphy PM. Chemokine receptor CX3CR1 mediates skin wound healing by promoting macrophage and fibroblast accumulation and function. The Journal of Immunology. 2008;180(1):569–579.

6. Schmidt BA, Horsley V. Intradermal adipocytes mediate fibroblast recruitment during skin wound healing. Development. 2013;140(7):1517–1527.

7. DiPietro LA, Burdick M, Low QE, Kunkel SL, Strieter RM, et al. MIP-1alpha as a critical macrophage chemoattractant in murine wound repair. The Journal of clinical investigation. 1998;101(8):1693–1698.

8. Low QE, Drugea IA, Duffner LA, Quinn DG, Cook DN, Rollins BJ, et al. Wound healing in MIP-1α-/- and MCP-1-/- mice. The American journal of pathology. 2001;159(2):457–463.

9. Egozi EI, Ferreira AM, Burns AL, Gamelli RL, Dipietro LA. Mast cells modulate the inflammatory but not the proliferative response in healing wounds. Wound Repair and Regeneration. 2003;11(1):46–54.

10. Dovi JV, He LK, DiPietro LA. Accelerated wound closure in neutrophil-depleted mice. Journal of leukocyte biology. 2003;73(4):448–455.

11. Neuman RE, Logan MA, et al. The determination of hydroxyproline. J Biol Chem. 1950;184(1):299–306.

12. Duck FA. Physical properties of tissues: a comprehensive reference book. Academic press; 2013.

13. Jimenez SA, Millan A, Bashey RI. Scleroderma-like alterations in collagen metabolism occurring in the TSK (tight skin) mouse. Arthritis & Rheumatism: Official Journal of the American College of Rheumatology. 1984;27(2):180–185.

14. Boyer B, Kern P, Fourtanier A, Labat-Robert J. Age-dependent variations of the biosyntheses of fibronectin and fibrous collagens in mouse skin. Experimental gerontology. 1991;26(4):375–383.

15. Yamamoto T, Takagawa S, Katayama I, Yamazaki K, Hamazaki Y, Shinkai H, et al. Animal model of sclerotic skin. I: Local injections of bleomycin induce sclerotic skin mimicking scleroderma. Journal of Investigative Dermatology. 1999;112(4):456–462.

16. Saito E, Fujimoto M, Hasegawa M, Komura K, Hamaguchi Y, Kaburagi Y, et al. CD19-dependent B lymphocyte signaling thresholds influence skin fibrosis and autoimmunity in the tight-skin mouse. The Journal of clinical investigation. 2002;109(11):1453–1462.

17. Markova MS, Zeskand J, McEntee B, Rothstein J, Jimenez SA, Siracusa LD. A role for the androgen receptor in collagen content of the skin. Journal of investigative dermatology. 2004;123(6):1052–1056.

18. Manne J, Markova M, Siracusa LD, Jimenez SA. Collagen content in skin and internal organs of the tight skin mouse: an animal model of scleroderma. Biochemistry research international. 2013;2013.

19. Tarnutzer K, Sankar DS, Dengjel J, Ewald CY. Collagen constitutes about twelve percent in females and seventeen percent in males of the total protein in mice. bioRxiv. 2022;.

20. Yang G, Crawford RC, Wang JH. Proliferation and collagen production of human patellar tendon fibroblasts in response to cyclic uniaxial stretching in serum-free conditions. Journal of biomechanics. 2004;37(10):1543–1550.
