# Supplementary material for: Mechano-biological and bio-mechanical pathways in cutaneous wound healing
Source: PLoS Comput Biol. 2023 Mar 9;19(3):e1010902. doi: 10.1371/journal.pcbi.1010902 (PMC10030043; doi:10.1371/journal.pcbi.1010902)
Supplement: S2 Appendix — (PDF) [file pcbi.1010902.s012.pdf]

S2 Appendix. Sensitivity of s_ρ to Ω^m for sub-physiological elastic stretch. In Fig. 7, we have shown that assuming a stretch-mediated mechanobiological coupling yields a marked decrease in ρ and other variables in the wound when increasing the strength of the coupling (Ω^m), a behavior that we have explained in terms of the sub-physiological elastic deformation in the wound that results from imposing an initially stress-free fibrin clot. Here, we further analyze the link between ρ and Ω^m by analytically computing the derivative of the source term in Eq. (6), s_ρ, with respect to the parameter defining the coupling strength, Ω^m:

$$\frac{\partial s_\rho}{\partial \Omega^m} = \rho\, p_{\rho,n} \left(1 - \frac{\rho}{K_{\rho,\rho}}\right) \hat{H}(\theta^e, \vartheta^{ph}, \gamma^e) - \rho \frac{\partial d_\rho}{\partial \Omega^m}, \tag{A2.1}$$

where d_ρ depends on Ω^m through the homeostasis constraint imposed considering an unwounded and physiologically-loaded tissue

$$d_\rho = p_{\rho,n} \left(1 + \frac{\Omega^b}{K_{c,c}+1} + \frac{\Omega^m}{2}\right) \left(1 - \frac{1}{K_{\rho,\rho}}\right), \tag{A2.2}$$

such that

$$\frac{\partial s_\rho}{\partial \Omega^m} = \frac{\rho\, p_{\rho,n}}{2} \left[2\hat{H}(\theta^e, \vartheta^{ph}, \gamma^e) \left(1 - \frac{\rho}{K_{\rho,\rho}}\right) - \left(1 - \frac{1}{K_{\rho,\rho}}\right)\right]. \tag{A2.3}$$

Since we are considering sub-physiological values of the elastic deformation, we can use the inequality $\hat{H}(\theta^e, \vartheta^{ph}, \gamma^e) < 1/2$ to write

$$\frac{\partial s_\rho}{\partial \Omega^m} < \frac{p_{\rho,n}}{2K_{\rho,\rho}} \rho\,(1-\rho), \tag{A2.4}$$

whose right-hand side term provides an upper bound for $\partial s_\rho / \partial \Omega^m$ and has sign depending on ρ, indicating that s_ρ will decrease for increasing Ω^m for values of $\rho \geq 1$ when $\theta^e < \vartheta^{ph}$.
