# Supplementary material for: Mechano-biological and bio-mechanical pathways in cutaneous wound healing
Source: PLoS Comput Biol. 2023 Mar 9;19(3):e1010902. doi: 10.1371/journal.pcbi.1010902 (PMC10030043; doi:10.1371/journal.pcbi.1010902)
Supplement: S3 Appendix — (PDF) [file pcbi.1010902.s013.pdf]

S3 Appendix. Equilibrium points for ODE system comprising Eqs. (7,7,18). In Fig. 8, we have observed that a sufficiently large value of Ω^m, *e.g.* $\Omega^m = 0.8$, induces sustained overexpression of ρ, ϕ_c, ξ_c, and k_1 throughout the simulated wound healing time span. To provide an explanation for this peculiar behavior, we study the equilibrium of the ODE system comprising Eqs. (6,7,18), where the definitions of k_1 and $\hat{H}$ are given in Eq. (17) and Eq. (19), respectively. This system of equations can be regarded as descriptive of a 0-dimensional tissue region lacking any biochemical field diffusion (Eq. 3) or ECM remodeling (Eq. (8)). Note that we exclude Eq. (5) from the ODE system, and thus set $c = 1$, corresponding to the assumption of a negligible contribution of the secondary inflammation wave on the long-term tissue behavior. Focusing on the region of space $\rho \times \phi_c \times \xi_c = [0; 8] \times [0; 3] \times [0; 3]$, we set the model parameters to the values indicated in S3 Table and S4 Table and identify the loci of points satisfying the conditions $s_\rho = 0$, $\dot{\phi}_c = 0$, and $\dot{\xi}_c$, respectively. More explicitly, we solve each of the following nonlinear algebraic equations for alternative choices of Ω^m, knowing that the system will be in equilibrium when all equations are satisfied:

$$\begin{aligned}
0 &= p_{\rho,n} \left(1 + \frac{\Omega^b_\rho}{K_{\rho,c}+1} + \Omega^m \, \hat{H}\right) \left(1 - \frac{\rho}{K_{\rho,\rho}}\right) \rho - d_\rho \, \rho \\
0 &= p_{\phi_c,n} \left(1 + \frac{\Omega^b_{\phi_c}}{K_{\phi_c,c}+1} + \Omega^m \, \hat{H}\right) \frac{\rho}{K_{\phi_c,\rho} + \phi_c} - (d_{\phi_c} + \rho \, d_{\phi_c,\rho,c}) \, \phi_c \\
0 &= \frac{p_{\xi_c,\phi_c}}{K_{\xi_c,\phi_c} + \xi_c} \, \phi_c - \left[d_{\xi_c} + d_{\xi_c,\dot{\phi}_c^{-}} \, (d_{\phi_c} + \rho \, d_{\phi_c,\rho,c}) \, \phi_c\right] \xi_c
\end{aligned} \tag{A3.1}$$

First, we consider the default value $\Omega^m = 0.01$. As shown in Fig. A3.1a, the surfaces corresponding to $s_\rho = 0$ (blue), $\dot{\phi}_c = 0$ (green), $\dot{\xi}_c = 0$ (red) intersect along multiple lines, but there are only 2 points where all of them are satisfied, *i.e.* where the system is in equilibrium. One is the trivial solution $(\rho, \phi_c, \xi_c) = (0, 0, 0)$, capturing the notion that a system without cells or collagen will not spontaneously accumulate any of these quantities nor crosslinks. Instead, the non-trivial solution $(\rho, \phi_c, \xi_c) = (1, 1, 1)$ corresponds to the physiological condition and will act as attractor for any tissue in supra-physiological conditions, indicating that the wound behavior shown in Fig. 8 for $\Omega^m = 0.01$ will eventually evolve towards the physiological state of the unwounded tissue. Similar observations hold for the case $\Omega^m = 0.4$, Fig. A3.1b. Conversely, when $\Omega^m = 0.8$, the system exhibits 3 non-trivial and 1 trivial equilibrium points (Fig. A3.1c). This phase-space topology implies a bi-stable system, with one stable point continuing to be the physiological equilibrium, and the other stable point corresponding to a fibrotic state. To confirm this, we consider the results of our FE simulations for $\Omega^m = 0.8$ and represent the streamlines on planes of constant ξ_c, ρ, or ϕ_c, both for the unwounded (far field) and wounded tissue at day 21 post-wounding. As visible in Fig. A3.1d–f, the equilibrium point at $(1, 1, 1)$ acts as an attractor for the far field (black dot), which will thus eventually return to the physiological state. On the contrary (Fig. A3.1g–i), the day-21 values of ρ, ϕ_c, and ξ_c place the wounded tissue (red dot) in a different landscape region, where the attractor is the point at $(\rho, \phi_c, \xi_c) = (6.1, 2.4, 1.7)$, leading the system towards a supra-physiological steady state that corresponds to permanent fibrosis.

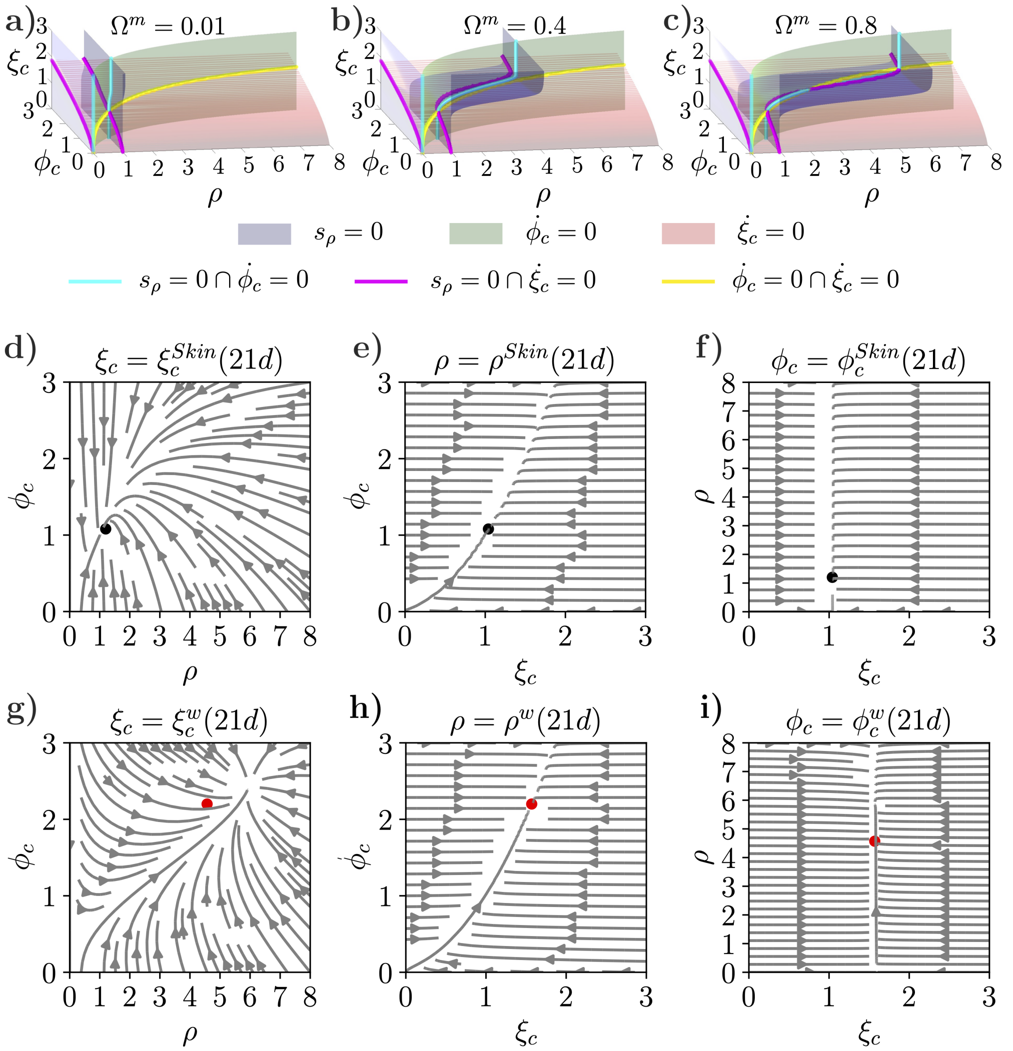

Figure A3.1. Stability analysis of an ODE system representative of long-term tissue progression. (**a–c**) Loci of points independently satisfying the conditions $s_\rho = 0$ (blue surface), $\dot{\phi}_c = 0$ (green surface), and $\dot{\xi}_c$ (red surface), as well as their intersections (cyan, magenta, and yellow lines). Line intersections identify equilibrium points for the ODE system. The phase-space topology, and thus the number of equilibrium points, is affected by the value of the coupling parameter Ω^m. (**d–f**) Streamlines on planes of constant ξ_c, ρ, or ϕ_c for unwounded skin tissue and $\Omega^m = 0.8$. The black dot indicates the state of skin at day 21 post-wounding, as obtained from FE simulations. (**g–i**) Streamlines on planes of constant ξ_c, ρ, or ϕ_c for wounded tissue and $\Omega^m = 0.8$. The red dot indicates the state of the wound at day 21 post-wounding, as obtained from FE simulations.
