# Supplementary material for: Mechano-biological and bio-mechanical pathways in cutaneous wound healing
Source: PLoS Comput Biol. 2023 Mar 9;19(3):e1010902. doi: 10.1371/journal.pcbi.1010902 (PMC10030043; doi:10.1371/journal.pcbi.1010902)
Supplement: S1 Table — (PDF) [file pcbi.1010902.s014.pdf]

S1 Table. Search ranges for Bayesian model parameters.

Model parameter	Search range
$\alpha_{C_{10}}$	$0.0001 - 1$ MPa
α_{k_1}	$0.1 - 1'000$ MPa
α_{k_2}	$0.1 - 1'000$
α_{Σ}	$0.0001 - 1$ MPa
$\sigma_{C_{10}}$	$0 - 2$
σ_{k_1}	$0 - 2$
σ_{k_2}	$0 - 2$
σ_{Σ}	$0 - 1$
