# Supplementary material for: Mechano-biological and bio-mechanical pathways in cutaneous wound healing
Source: PLoS Comput Biol. 2023 Mar 9;19(3):e1010902. doi: 10.1371/journal.pcbi.1010902 (PMC10030043; doi:10.1371/journal.pcbi.1010902)
Supplement: S2 Table — (PDF) [file pcbi.1010902.s015.pdf]

S2 Table. Global simulation parameters.

Parameter	Value	Source
Total simulation time	504 h = 21 d	From range of available data
Simulation time step	0.05 h	Sufficiently small to ensure convergence of the remodeling algorithm
Domain size in X: L_X	50 mm	$10D^w$
Domain size in Y: L_Y	50 mm	$10D^w$
Wound diameter: D^w	5.0 mm	Ref. [1]
Skin thickness	1.7 mm	Ref. [2]
Physiological stretch along x: λ_x	1.15	Within range from Refs. [3–5]
Physiological stretch along y: λ_y	1.15	Within range from Refs. [3–5]
Physiological value of θ^e: ϑ^{ph}	1.3225	$= \lambda_x \lambda_y$

References

1. Pensalfini M, Haertel E, Hopf R, Wietecha M, Werner S, Mazza E. The mechanical fingerprint of murine excisional wounds. Acta Biomaterialia. 2018;65:226–236.

2. Wietecha MS, Pensalfini M, Cangkrama M, Müller B, Jin J, Brinckmann J, et al. Activin-mediated alterations of the fibroblast transcriptome and matrisome control the biomechanical properties of skin wounds. Nature communications. 2020;11(1):1–20.

3. Dauendorffer J, Bastuji-Garin S, Guéro S, Brousse N, Fraitag S. Shrinkage of skin excision specimens: formalin fixation is not the culprit. British Journal of Dermatology. 2009;160(4):810–814.

4. Upchurch DA, Malenfant RC, Wignall JR, Ogden DM, Saile K. Effects of sample site and size, skin tension lines, surgeon, and formalin fixation on shrinkage of skin samples excised from canine cadavers. American journal of veterinary research. 2014;75(11):1004–1009.

5. Meador WD, Sugerman GP, Story HM, Seifert AW, Bersi MR, Tepole AB, et al. The regional-dependent biaxial behavior of young and aged mouse skin: A detailed histomechanical characterization, residual strain analysis, and constitutive model. Acta biomaterialia. 2020;101:403–413.
