# Supplementary material for: Mechano-biological and bio-mechanical pathways in cutaneous wound healing
Source: PLoS Comput Biol. 2023 Mar 9;19(3):e1010902. doi: 10.1371/journal.pcbi.1010902 (PMC10030043; doi:10.1371/journal.pcbi.1010902)
Supplement: S3 Table — (PDF) [file pcbi.1010902.s016.pdf]

S3 Table. Parameters for diffusible biochemical fields.

Parameter	Value	Eq.	Source
$D_{\alpha,\alpha}$: diffusion coefficient for 1st inflammatory wave	0.075 mm^2/h	3	Assumed $= D_{c,c}$
d_α: decay coefficient for 1st inflammatory wave	0.01 1/h	4	Assumed $= d_c$
$D_{c,c}$: diffusion coefficient for 2nd inflammatory wave	0.075 mm^2/h	3	Ref. [1]
d_c: decay coefficient for 2nd inflammatory wave	0.01 1/h	5	Ref. [1]
$p_{c,\alpha}$: effect of 1st inflammation on 2nd inflammation	0.23 1/h	5	Selected to match peak in c
$p_{c,\rho}$: effect of cell proliferation on 2nd inflammation	1.5 1/h	5	Ref. [2]
c_{tgt}: attractor for 2nd inflammation	1.01	5	From c homeostasis ($s_c = 0$)
$K_{c,c}$: self-saturation of 2nd inflammatory wave	0.5	5	Assumed
$D_{\rho,\rho}$: diffusion coefficient for cell population	0.035 mm^2/h	3	Ref. [1]
$D_{\rho,c}$: chemotactic diffusion coefficient for cells	8×10^{-5} mm^2/h	3	Ref. [1]
$p_{\rho,n}$: baseline cell mitotic rate for $c = 0$ and $\hat{H}_\rho = 0$	0.034 1/h	6	Ref. [1]
Ω^b_ρ: enhanced cell proliferation due to inflammation	5	6	Selected to match peak in ρ
Ω^m_ρ: enhanced cell proliferation due to mechanics	0.01<br>(Range: $0 - 0.8$)	6	Ref. [3], varied in Figs. 7-8
d_ρ: decay coefficient for cell population	0.048 1/h<br>($0.048 - 0.061$ 1/h)	6	From ρ homeostasis ($s_\rho = 0$)
γ^e: slope of the function $\hat{H}(\theta^e, \vartheta^{ph}, \gamma^e)$	5	10	Ref. [1]
γ^{k_1}: slope of the function $\hat{H}(c, k_1^c, k_1^{ph}, \gamma^{k_1})$	0.016	19	Assumed
$K_{\rho,\rho}$: self-saturation of cell population	30	6	Selected to match peak in ρ
$K_{\rho,c}$: saturation of cell population due to inflammation	10	6	Selected to match peak in ρ
$f_{\rho,n}$: baseline active tension for $\rho = 1$ and $c = 0$	0.04 MPa/mm^3	11	Ref. [1]
$K_{f,c}$: saturation of active tension due to inflammation	10^{-5}	11	Ref. [1]
Ω^b_f: increase of active tension due to inflammation	1	11	Ref. [1]

References

1. Buganza Tepole A. Computational systems mechanobiology of wound healing. Computer Methods in Applied Mechanics and Engineering. 2017;314:46–70.

2. Li D, Lee S, Tseng S. Differential expression and regulation of TGF-beta1, TGF-beta2, TGF-beta3, TGF-betaRI, TGF-betaRII and TGF-betaRIII in cultured human corneal, limbal, and conjunctival fibroblasts. Current eye research. 1999;19(2):154–161.

3. Yang G, Crawford RC, Wang JH. Proliferation and collagen production of human patellar tendon fibroblasts in response to cyclic uniaxial stretching in serum-free conditions. Journal of biomechanics. 2004;37(10):1543–1550.
