# Supplementary material for: Mechano-biological and bio-mechanical pathways in cutaneous wound healing
Source: PLoS Comput Biol. 2023 Mar 9;19(3):e1010902. doi: 10.1371/journal.pcbi.1010902 (PMC10030043; doi:10.1371/journal.pcbi.1010902)
Supplement: S4 Table — (PDF) [file pcbi.1010902.s017.pdf]

S4 Table. Parameters for microstructural fields.

Parameter	Value	Eq.	Source
$p_{\phi_c,n}$: rate of collagen deposition for $c = 0$ and $\hat{H}_{\phi_c}$	0.002 1/h	7	Ref. [1]
$\Omega^b_{\phi_c}$: enhanced ϕ_c deposition due to cell proliferation	2.5	7	Selected to match peak in ϕ_c
$\Omega^m_{\phi_c}$: enhanced ϕ_c deposition due to mechanics	$= \Omega^m_\rho$	7	Assumed
$K_{\phi_c,c}$: saturation of collagen due to inflammation	10^{-4}	7	Ref. [1]
$K_{\phi_c,\rho}$: saturation of collagen due to cell proliferation	1.06	7	Ref. [1]
d_{ϕ_c}: decay coefficient for collagen content	0.00292 1/h<br>(0.00291 − 0.00330 1/h)	7	From ϕ_c homeostasis ($\dot{\phi}_c = 0$)
$d_{\phi_c,\rho,c}$: rate of collagen degradation due to ρ and c	4.85×10^{-4} 1/h	7	Ref. [1]
p_{ξ_c,ϕ_c}: natural forward rate of collagen crosslinking	1 1/h	18	Assumed
K_{ξ_c,ϕ_c}: saturation of crosslinking in response to ϕ_c	0.5	18	Assumed
d_{ξ_c}: decay coefficient for collagen crosslinking	0.66326 1/h<br>(0.66288 − 0.66327 1/h)	18	From ξ_c homeostasis ($\dot{\xi}_c = 0$)
$d_{\xi_c,\dot{\phi}_c^-}$: reduction of crosslinking due to ϕ_c depletion	1	18	Assumed
a: nonlinearity of the relation between k_1 and ξ_c	10	17	Selected to match k_1 evolution
$d_{\phi^w_f}$: decay coefficient for wound fibrin content	$= d_{\phi_c}$	15	Assumed
$d_{\phi^w_f,\rho,c}$: rate of fibrin degradation due to ρ and c	0.00325 1/h	15	To have negligible fibrin by d7
τ_{λ^p}: time constant for tissue growth	0.485	8	Ref. [1]

References

1. Buganza Tepole A. Computational systems mechanobiology of wound healing. Computer Methods in Applied Mechanics and Engineering. 2017;314:46–70.
