# Supplementary material for: Mechano-biological and bio-mechanical pathways in cutaneous wound healing
Source: PLoS Comput Biol. 2023 Mar 9;19(3):e1010902. doi: 10.1371/journal.pcbi.1010902 (PMC10030043; doi:10.1371/journal.pcbi.1010902)
Supplement: S5 Table — (PDF) [file pcbi.1010902.s018.pdf]

S5 Table. Emergent constitutive biomechanical parameters (Eqs. (2,16,17)).

Parameter	Value	Source
C_{10}^{Skin}: value for unwounded skin	12 kPa	Median from experiment fitting, Fig. 4a
k_1^{Skin}: value for unwounded skin	1.6 MPa	Median from experiment fitting Fig. 4b
k_2^{Skin}: value for unwounded skin	0.88 MPa	Median from experiment fitting Fig. 4c
$C_{10}^{i.w.}$: value for just-wounded tissue	0.0 kPa	Due to tissue removal as part of wounding
$k_1^{i.w.}$: value for just-wounded tissue	0.0 MPa	Due to tissue removal as part of wounding
$C_{10}^{Clot,\, d0}$: value for a freshly-formed clot	300.0 kPa	Assumed in line with Ref. [1]
$C_{10}^{d0,\, lb}$: lower bound for a d0 wound	2.5 kPa	Linear extrapolation from known d7 and d14 values
$C_{10}^{d7,\, lb}$: lower bound for a d7 wound	1.2 kPa	2.5^{th} percentile from experiment fitting, Fig. 4a
$C_{10}^{d14,\, lb}$: lower bound for a d14 wound	0.0 kPa	2.5^{th} percentile from experiment fitting, Fig. 4a
$k_1^{d0,\, lb}$: lower bound value for d0 wound	0.0 MPa	$= k_1^{i.w.}$ since no collagen is deposited prior to d0
$k_1^{d7,\, lb}$: lower bound value for d7 wound	0.6 MPa	2.5^{th} percentile from experiment fitting, Fig. 4b
$k_1^{d14,\, lb}$: lower bound value for d14 wound	1.2 MPa	2.5^{th} percentile from experiment fitting, Fig. 4b
$C_{10}^{d0,\, m}$: median value for d0 wound	51.2 kPa	Linear extrapolation from known d7 and d14 values
$C_{10}^{d7,\, m}$: median value for d7 wound	25.9 kPa	50th percentile from experiment fitting, Fig. 4a
$C_{10}^{d14,\, m}$: median value for d14 wound	0.6 kPa	50th percentile from experiment fitting, Fig. 4a
$k_1^{d0,\, m}$: median value for d0 wound	0.0 MPa	$= k_1^{i.w.}$ since no collagen is deposited prior to d0
$k_1^{d7,\, m}$: median value for d7 wound	19.0 MPa	50th percentile from experiment fitting, Fig. 4b
$k_1^{d14,\, m}$: median value for d14 wound	9.0 MPa	50th percentile from experiment fitting, Fig. 4b
$C_{10}^{d0,\, ub}$: upper bound value for d0 wound	167.2 kPa	Linear extrapolation from known d7 and d14 values
$C_{10}^{d7,\, ub}$: upper bound value for d7 wound	94.3 kPa	97.5^{th} percentile from experiment fitting, Fig. 4a
$C_{10}^{d14,\, ub}$: upper bound value for d14 wound	21.4 kPa	97.5^{th} percentile from experiment fitting, Fig. 4a
$k_1^{d0,\, ub}$: upper bound value for d0 wound	0.0 MPa	$= k_1^{i.w.}$ since no collagen is deposited prior to d0
$k_1^{d7,\, ub}$: upper bound value for d7 wound	86.2 MPa	97.5^{th} percentile from experiment fitting, Fig. 4b
$k_1^{d14,\, ub}$: upper bound value for d14 wound	148.1 MPa	97.5^{th} percentile from experiment fitting, Fig. 4b

References

1. Lee YU, Lee A, Humphrey J, Rausch M. Histological and biomechanical changes in a mouse model of venous thrombus remodeling. Biorheology. 2015;52(3):235–245.
